# Abiotic Stress and Reactive Oxygen Species: Generation, Signaling, and Defense Mechanisms

**DOI:** 10.3390/antiox10020277

**Published:** 2021-02-11

**Authors:** Swati Sachdev, Shamim Akhtar Ansari, Mohammad Israil Ansari, Masayuki Fujita, Mirza Hasanuzzaman

**Affiliations:** 1 Department of Environmental Science, School for Environmental Sciences, Babasaheb Bhimrao Ambedkar University, Vidya Vihar, Rae Bareli Road, Lucknow 226 025, India; swati_sachdev2003@yahoo.com; 2 Institute of Forest Research and Productivity, Ranchi 835 303, India; shamimansari_1@yahoo.co.uk; 3 Department of Botany, University of Lucknow, Lucknow 226 007, India; 4 Laboratory of Plant Stress Responses, Department of Applied Biological Science, Faculty of Agriculture, Kagawa University, 2393 Ikenobe, Miki-cho, Kita-gun, Kagawa 761-0795, Japan; 5 Department of Agronomy, Faculty of Agriculture, Sher-e-Bangla Agricultural University, Dhaka 1207, Bangladesh

**Keywords:** abiotic stress, antioxidant, biomolecules, climate change, reactive oxygen species

## Abstract

Climate change is an invisible, silent killer with calamitous effects on living organisms. As the sessile organism, plants experience a diverse array of abiotic stresses during ontogenesis. The relentless climatic changes amplify the intensity and duration of stresses, making plants dwindle to survive. Plants convert 1–2% of consumed oxygen into reactive oxygen species (ROS), in particular, singlet oxygen (^1^O_2_), superoxide radical (O_2_^•–^), hydrogen peroxide (H_2_O_2_), hydroxyl radical (^•^OH), etc. as a byproduct of aerobic metabolism in different cell organelles such as chloroplast, mitochondria, etc. The regulatory network comprising enzymatic and non-enzymatic antioxidant systems tends to keep the magnitude of ROS within plant cells to a non-damaging level. However, under stress conditions, the production rate of ROS increases exponentially, exceeding the potential of antioxidant scavengers instigating oxidative burst, which affects biomolecules and disturbs cellular redox homeostasis. ROS are similar to a double-edged sword; and, when present below the threshold level, mediate redox signaling pathways that actuate plant growth, development, and acclimatization against stresses. The production of ROS in plant cells displays both detrimental and beneficial effects. However, exact pathways of ROS mediated stress alleviation are yet to be fully elucidated. Therefore, the review deposits information about the status of known sites of production, signaling mechanisms/pathways, effects, and management of ROS within plant cells under stress. In addition, the role played by advancement in modern techniques such as molecular priming, systems biology, phenomics, and crop modeling in preventing oxidative stress, as well as diverting ROS into signaling pathways has been canvassed.

## 1. Introduction

Climate change has drastically reduced the environmental services, enhancing plants’ vulnerability to various abiotic stresses during ontogenesis [1] that disparages their struggle for survival, growth, and economic output [2]. Abiotic stresses encompassing heat shock, chilling/freezing, water-deficit, waterlogging, salinity, nutrient imbalance, heavy metals, and xenobiotic stress account for 50% productivity loss [3]. The contributory environmental factors are extreme temperature events (low or high), excess irradiation (UV-A and UV-B), fluctuation in light intensities (low or high), strong storm events, non-uniformity in the rainfall pattern (deficit or excess), discharge and accumulation of heavy metals, and other xenobiotic compounds (pesticides, fertilizers, hydrocarbons) [4,5,6]. In a natural environment, the abiotic stresses often occur in combination [7] due to their interrelated pathways and show unparalleled and compounded effects on plants, impinging their cellular, metabolic, and physiological activities [1,5].

Reactive oxygen species (ROS) such as superoxide radical (O_2_**^•^**^–^), hydrogen peroxide (H_2_O_2_), hydroxyl radical (^•^OH), singlet oxygen (^1^O_2_), peroxy radical (ROO**^•^**), and alkoxyl radicals (RO**^•^**) are produced at low temperature within a threshold concentration in the plant cell under ambient environmental conditions. However, the extreme environmental conditions trigger excessive production of ROS [8]. ROS damage molecular and cellular components due to the oxidation of biomolecules (lipid, carbohydrates, proteins, enzymes, DNA) and cause plant death [6,9]. To avert the damages, plants tightly regulate ROS production via the recruitment of enzymatic and non-enzymatic antioxidants. The enzymatic antioxidant system comprising superoxide dismutase (SOD), catalase (CAT), ascorbate peroxidase (APX), glutathione reductase (GR), peroxidase (POX), etc. and non-enzymatic antioxidants such as vitamins, flavonoids, stilbenes, and carotenoids quench the excess ROS, thereby providing a shield against oxidative stress [7,10,11]. Unfettered propagation of oxygen (O_2_) derived reactive species is detrimental to the plant health. However, a controlled ROS production participates in redox signaling, plant growth, and development during stress [12]. Fine-tuned ROS production mediates cell to cell communication by magnifying signals via the nicotinamide adenine dinucleotide phosphate (NADPH) oxidase, also called respiratory burst oxidase homolog (RBOH) and retaliating stress by modulating the protein structure and activating defense responsive genes [9].

The occurrence of abiotic stresses either individually or simultaneously triggers the overproduction of ROS in plant cells that becomes a major challenge for optimal plant growth and productivity. Exploring the underlying molecular mechanisms of ROS signaling pathways assumes a great significance to mitigate stress, or promote signaling under current and future climatic scenarios, as well as retain tolerance and economic productivity in plants of economic importance. The present review provides a critical analysis of the accumulated knowledge on the impact of plant fitness under abiotic stresses as well as explores antioxidant-based defense mechanisms regulating ROS accumulation and dissipating oxidative stress. The review unravels the dual role of ROS as a signaling molecule triggering plant acclimatization and development under stress(es). Moreover, the implication of scientific and technological applications such as molecular priming, systems biology, phenomics, and crop modeling to fortify plants’ tolerance against oxidative stress has also been discussed.

## 2. Climate Change Triggers Abiotic Stress and ROS Generation

**Figure 1:**
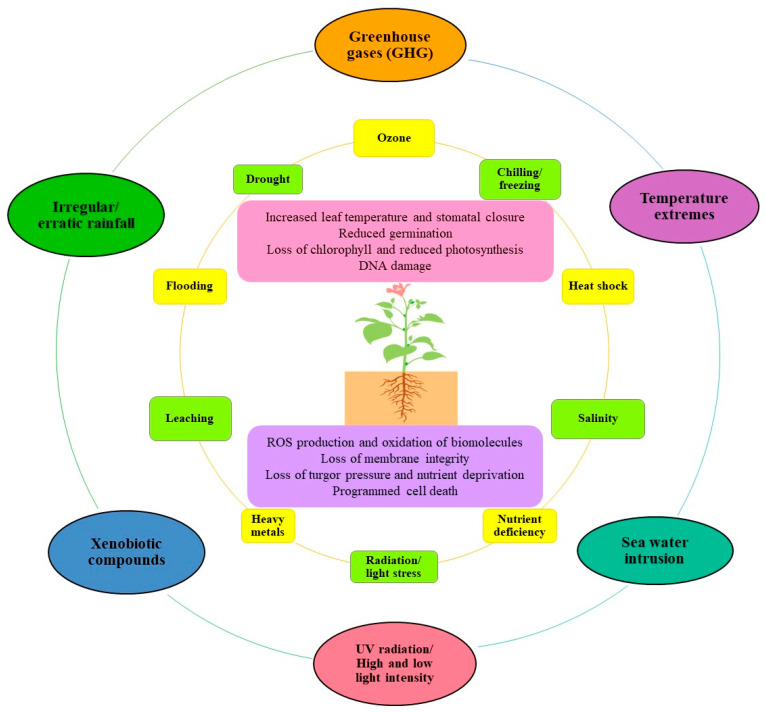
Climate change intensifies the magnitude of abiotic stresses that severely affect plant growth and physiological activities. Various abiotic factors modified by environmental conditions (outer circle) leads to abiotic stresses (the inner circle) that hamper plant physiological and metabolic activities, biomolecules, cellular structure, growth, and productivity (rectangular innermost blocks).

Climate change has escalated the prevalence of abiotic stress and their debt on plants (Figure 1), which is witnessed on a broad geographical scale. FAO (2019) has reported that 96.5% of the global cultivation area experiences one or the other kind of stress [13]. The atmospheric enrichment of greenhouse gases has raised the mean global surface temperature (0.85 °C from 1880 to 2012) and changed rainfall patterns [14]. It has been anticipated that a 1 °C hike in temperature enhances 4–4.5% more water requirement in plants [9,15] making plant cultivation a more denting task in drought-affected areas. An increase in temperature is suspected to bio-transform chemical pollutants into more toxic or bioactive forms that will aggravate environmental nuisance and perniciously affect plant homeostasis [16]. A rise in temperature coupled with precipitation reduction promotes the volatilization of xenobiotic compounds as persistent organic pollutants, exacerbating air pollution. On the other hand, the excess precipitation enhances deposits of air pollutants on land and reinforces the leaching of soil nutrients and pollutants to groundwater causing soil pollution, aquifer contamination, nutrient imbalance, and salinity [16,17,18]. In normal circumstances, fluctuation in temperature and rainfall, nutrient imbalance, waterlogging, etc. temporarily and competitively restrict plant growth. However, due to extreme climatic events and fluctuations in routine weather conditions, both the severity and duration of stresses prolong and get amplified, drastically eclipsing the plant performance beyond recovery [13,19]. Climate change has been geared up to create adverse conditions that plants cannot escape and face several vandalizing impacts of abiotic stresses (Table 1). Improving plant growth and productivity to feed the existing global population is not the only challenge, but to fulfill the nutritional needs of the future generation is equally important. Therefore, it is crucial to review the extent of impinging effects of various persisting abiotic stresses on plants. Coupling these data with simulation models could help chalk out sustainable strategies for crop protection in accordance with the projected change in environmental conditions.

### 2.1. Temperature Stress

The extreme variation in temperature (10–15 °C deviation) above or below an optimum condition induces heat or chilling/freezing stress that impairs photosynthesis, plant architecture, reproduction, and productivity [37]. A plant encountered with heat stress undergoes morphological, cellular, and metabolic changes that decrease the function of photosynthetic and respiratory apparatus, reduce enzymatic activity, upregulate transcription, and translation of heat shock proteins (HSP), increase calcium (Ca^2+^) influx, and intensify ROS production [37]. Heat stress inhibits the cell differentiation process, therefore, affecting the leaf area [38]. Exposure of hyacinth bean (*Lablab purpureus* L.) to a high temperature significantly affects membrane permeability, increases ROS production, and lipid peroxidation; abridges plant growth, productivity, and leaf area; reduces leaf chlorophyll and carotenoid content; and causes an imbalance between the generation and scavenging of H_2_O_2_ and O_2_**^•^**^–^ [39]. The imposition of cucumber plant to heat stress reduces growth, yield, chlorophyll content, photosynthesis, stomatal conductance, transpiration rate, antioxidants, and membrane stability index, while increasing ROS production, lipid peroxidation, intercellular carbon dioxide (CO_2_) concentration, and non-photochemical quenching (NPQ) [38]. A high temperature elevates the production of ozone (O_3_) in the troposphere which imposes oxidative stress on plants [40]. Chilling stress characterized by low-temperature events facilitates solubility and the accumulation of O_2_ and electron leakage from the photosynthetic electron transport chain (ETC)/reduction of respiratory ETC that together enhances ROS production in plant cells [41], affecting membrane fluidity and enzymes activities [42]. Under chilling stress, an enhanced electrolyte leakage with reduced chlorophyll and tissue water content has been reported in cucumber seedlings [43]. Increased malondialdehyde (MDA) content, *RBOH1* expression, and accumulation of H_2_O_2_ and O_2_**^•^**^–^ in leaves, and reduced net photosynthesis rate, as well as chlorophyll fluorescence, has been observed in tomato under low-temperature stress [44].

### 2.2. Water Stress

During the last decades, change in climatic scenarios has tremendously affected the rainfall patterns causing erratic precipitation with an altered magnitude and seasonal variations [45]. The situation fosters extremes of drought and flooding in different parts of the globe.

#### 2.2.1. Water Deficit (Drought)

Drought imposing water deficit stress leads to water scarcity, restricted growth, and yield in plants [23,46,47]. Water deficit stress sets a reduction in the plant water potential and turgor to the level that impairs the normal functioning of cells [45]. The physiological impact of water deficit conditions varies with the severity and duration of stress. Water deficit stress reduces stomatal opening, abridges CO_2_ fixation, accelerates photoreduction of O_2_ in the chloroplast, and increases photorespiration, eventually leading to ROS accumulation and oxidative damage in plants [42]. The reduced number of spells coupled with a high temperature has aggravated drought conditions in many parts of the world. According to a World Bank report (2006), India ranks second among the most severely drought-affected Asian countries [48]. Due to drought, worldwide productivity has reduced by 21% in wheat and 40% in maize during the past few years [23]. Lee et al. [49] have reported a decrease in dry mass, enhanced accumulation of ROS, and increased MDA content in white clover leaves under water deficit conditions.

#### 2.2.2. Waterlogging and Flooding

The excessive accumulation of water in soil due to heavy precipitation over a period of time, poor drainage, etc. causes soil flooding or waterlogging [50]. Nearly 10% of the world’s total land has been detrimentally affected by waterlogging [51]. During 2006–2016, two-thirds of the total global crop loss and damage has been attributed to floods [50]. Waterlogging covers plant roots and is characterized by low light, impaired gaseous exchange, hypoxia, and anoxia [50]. It reduces O_2_ diffusion by 10,000 times compared to air, thereby suppressing aerobic activity, including root respiration in soil [52]. The anoxic condition inhibits ETC of chloroplast and mitochondria that consequently results in the production of ROS [53,54]. Sesame plants subjected to waterlogging conditions show increased lipid peroxidation, ROS accumulation, and methylglyoxal content that induce oxidative stress [55]. In the case of clear flooded water, light easily reaches the submerged plant parts and induces photorespiration, and produces peroxisomal H_2_O_2_ [54]. Flooding also leaches out essential nutrients from the soil, accumulates salts, and increases the availability of heavy metals owing to the change in soil pH. These adverse changes ultimately induce nutrient deficiency and other stresses (salinity, heavy metal) in plants [56].

### 2.3. Salt Stress

Soil salinity has globally degraded nearly 20% of total arable and 33% of the irrigated land [57]. Excess sodium (Na^+^) and chloride (Cl^−^) ions present in the saline soil are transported and accumulated to the toxic level at the expense of other essential ions in plant cells [36]. Salt stress plants desisting water absorption experience drought-like conditions [36]. Therefore, salinity reduces stomatal conductance and disrupts photosystem (PS) and photosynthetic enzymes that lead to ROS production in plants [57]. The accumulation of ROS in plant cells under salinity is also mediated through the plasma membrane NADPH oxidase and apoplast (all parts beyond the plasma membrane including the cell wall) diamine oxidases (DAOs) [58]. The exposure of wheat cultivars to salinity stress increases ROS accumulation that induces lipid peroxidation and electrolyte leakage thereby reducing membrane stability [59]. The effects of salinity have been more pronounced on sensitive wheat cultivar HD2329. Similarly, the higher H_2_O_2_ accumulation and MDA content under salinity stress have been reported in a salt-sensitive cultivar of *Brassica juncea* as compared to its tolerant cultivar [60].

### 2.4. Nutrient Deficiency

Accessibility to essential nutrients, ensuring proper plant growth and development, has become a major challenge owing to the persistently changing attributes of global climate. The scarcity of essential plant nutrients in soil adversely affects their physiological activities particularly ETC, water relation, and gaseous exchange that contribute to ROS production and trigger oxidative stress in plants [61]. Plasma membrane-bounded NADPH oxidase is one of the major sources of ROS generation in plant cells [62]. Plant nutrients such as zinc (Zn^2+^) and potassium (K^+^) regulate the activity of NADPH oxidase and therefore, their scarcity elevates the enzyme activity which catalyzes the production of O_2_**^•^**^–^ [61] or H_2_O_2_ [63]. Nutrient starved plants elicit ROS production via the ethylene signaling cascade. The low availability of K^+^ prompts ethylene biosynthesis that, in turn, up-streams ROS production [64]. Mineral nutrients such as nitrogen (N), magnesium (Mg), copper (Cu), manganese (Mn), Zn, etc. are an integral part of various enzymes (Cu/Zn-SOD, Mn-SOD, etc.) and antioxidants that participate in energy metabolism or scavenge ROS [61,63,65]. The deficiency of nutrients impairs the ROS scavenging capacity of plants and indirectly results in ROS production [61,65]. For instance, the diminished potential of enzymes to scavenge H_2_O_2_ and O_2_**^•^**^–^ within plant cells increases the level of ^•^OH via the Heber-Weiss reaction [61]. Further deprivation of elements such as Mg which is a major constituent of chlorophyll impairs the photosynthetic activity resulting in ROS generation [66].

### 2.5. Heavy Metal and Xenobiotics Stress

The accumulation of non-essential metals shows toxicity in plants via ROS generation. However, the unrestricted uptake of essential nutrients also induces ROS production [32]. Heavy metals such as iron (Fe), chromium (Cr), and Cu are major redox-active metals that impose oxidative stress in plants owing to their high concentrations in soil [67]. Heavy metal stress triggers ROS production mediated through ETC of chloroplast, mitochondria, apoplast, and peroxisome [68,69]. Cadmium (Cd) is a non-essential metal that causes toxicity in plants. Cd supersedes Cu or Fe ions in antioxidant metalloenzymes with their impeded activities, indirectly inducing ROS production, impairing respiratory ETC, and interfering with the redox status in cells [63]. Despite being an essential micronutrient, the excess accumulation of Fe also initiates the production of ROS in plants through a series of reactions [70] and causes damage to the lipid membrane and chlorophyll [61]. The reduced form of Fe oxidizes to produce H_2_O_2_ and O_2_^•–^. In turn, H_2_O_2_ oxidizes the reduced Fe compounds to generate highly toxic ^•^OH [61]. This auto-oxidation of redox-active metals such as Fe and Cu consequently results in ROS formation, mediated by the Fenton-type reaction [67]. Homologous to heavy metals, xenobiotic compounds such as pesticides also trigger ROS production leading to oxidative stress [71]. Out of the total pesticides applied, only 1% reaches the target, the remaining very large proportion accumulates in soil and non-target living organisms [72]. Pesticides retard plant growth, abridge photosynthetic efficiency, induce molecular alterations, increase ROS production, and modify the antioxidant status [71,73]. The degradation of chlorophyll with an increase in H_2_O_2_ and MDA level has been reported in tomato leaves treated with thiram [73]. In another study, imidacloprid declines the chlorophyll content in *B. juncea* seedlings. The reduction in chlorophyll is attributed to an enhanced expression of gene *CHLASE* encoding chlorophyllase enzyme that catalyzes chlorophyll degradation [71]. Moreover, insecticides enhance the RBOH transcript level and ROS accumulation.

### 2.6. Co-Occurrence of Multiple Abiotic Stresses

Plants growing in natural conditions are exposed to multiple stresses at the same time. For example, an increase in temperature enhances evapotranspiration that induces stresses of water-deficit and soil salinization simultaneously and has a dramatic impact on growth and productivity. A combination of abiotic stress induces a unique and complex set of responses at the physiological, metabolic, and molecular levels, which are different than what is being observed under individual stress scenarios [28]. The confluence of heat and drought stress induces the closure of stomata, whereas the individual heat stress effect prompts the opening of stomata for transpiration and assists cooling in *Arabidopsis* [74]. Rizhsky et al. [75] have demonstrated differential physiological responses during heat shock, drought, and combined stress (heat+drought) in the tobacco plant. Drought reduces the respiration rate and photosynthesis, whereas heat shock increases the respiration rate without a significant change in the photosynthesis as compared to the control. The combined stress treatment reduces the process of photosynthesis compared to the individual drought stress but significantly enhances respiration compared to the heat shock stress. The stomatal conductance and leaf temperature significantly alter during the combined stress conditions. Stomatal conductance gets reduced and the leaf temperature, increased by 2–3 °C in plants, is exposed to stress combination due to the closed stomata and negligible transpiration. Analogously, Semwal and Khanna-Chopra [76] have reported that jointly operating heat and water deficit stress leads to ROS production, oxidative damage, and attenuates the antioxidant defense capacity (CAT activity and higher redox pool) in *Chenopodium album*.

Correspondingly, the combined stress conditions also provoke a dissimilar alteration at the molecular level in many cases. For instance, the individual gene in the *Arabidopsis* ROS gene network follows differential expressions under dissimilar stresses [75] due to different sets of responses being required under various stress conditions. As a result, the combination of stresses shows an independent and unique set of responses [77]. On exposure to the combined stresses of heat and drought, 770 specific transcripts have been recorded compared to the individual stress of either heat or drought, indicating elicitation of a unique acclimation response under stress combination [74]. Similarly, the combined effect of heat, drought, and biotic (viruses) stress induce molecular reprogramming leading to a significant reorganization of defense response [78]. Plants, to survive under the persisting combination of environmental cues, tailor their defense responses resulting in a cross-talk between various mechanisms. Several studies highlight that the cross-talks of regulatory molecules with signaling pathways trigger tolerance to multiple stresses [79]. The concurrent occurrence of stresses may have complementary or detrimental consequences on plants [76]. For example, in comparison to individual stress, the combined episode of heat and drought stress induces detrimental effects on physiological activities, growth, and productivity of several crops (maize, barley, sorghum) and grasses such as bluegrass [77]. It is difficult to predict the strategies adopted by plants to cope with the concert of diverse environmental stresses due to their tailored responses. However, the elucidation of cross-talk mechanisms among cellular pathways responsible for differential responses of various plant species under the concert of stresses can augment crop breeding programs to develop tolerant varieties.

## 3. Abiotic Stress-Induced Oxidative Stress in Cellular Compartments

**Figure 2:**
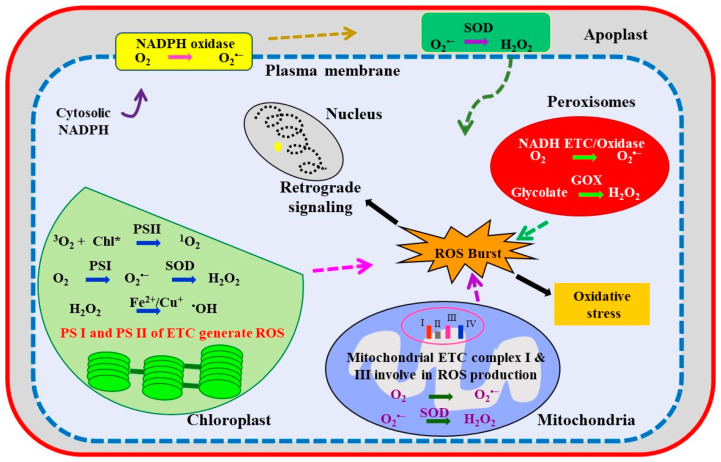
Abiotic stresses induced the production of ROS in different plant cell organelles which either initiate signaling (retrograde) or cause oxidative stress. In chloroplast singlet oxygen (^1^O_2_), superoxide radical (O_2_^•–^), hydrogen peroxide (H_2_O_2_), and hydroxyl radical (^•^OH) are produced by an excited chlorophyll (Chl*), via the electron transport chain (ETC) at PS I and II (Mehler’s reaction), dismutation of O_2_**^•^**^–^ by superoxide dismutase (SOD) and via the Fenton reaction catalyzed by reduced iron (Fe^2+^) and copper (Cu^+^), respectively. At peroxisomes, photorespiration (glycolate), enzymes, and NADH (nicotinamide adenine dinucleotide) dependent small ETC induce the production of O_2_**^•^**^–^ and H_2_O_2_. Mitochondrial ETC participates in the generation of O_2_**^•^**^–^ which on dismutation by SOD produces H_2_O_2_. Cytosolic NADPH induces conversion of O_2_ into O_2_**^•^**^–^ by the action of NADPH oxidase of the plasma membrane which further dis-mutates into H_2_O_2_ in the apoplast by SOD. ROS produced in different cell organelles under the duress of abiotic stresses mediate signaling pathways at a low/moderate concentration or induce oxidative stress at a high concentration.

Oxidative stress is an unparalleled and intricate phenomenon of imbalance in cellular redox homeostasis that arises due to an exponential increase in ROS [80]. Under stress conditions, the activity of antioxidants declines to aid in ROS accumulation at an uncompensated level, leading to oxidative burst and oxidative damage [81]. The generation of a particular ROS in a cell is highly localized and regulated by a particular compartment depending upon the operating enzymatic and non-enzymatic pathways [82,83]. The photosynthetic and respiratory ETC, plasma membrane-localized NADPH oxidases, and apoplast POXs are major pathways, which are mainly involved in ROS production in the plant cell [82]. The major events leading to ROS production in a plant cell under the influence of unfavorable abiotic conditions trigger either retrograde signaling or oxidative burst (Figure 2). ROS generated in different organelles affect ETC, chlorophyll, proteins, and enzymes. However, inducing a mechanism that curbs ROS at the initial point of generation in cell organelles can prevent further damage. Additionally, channelizing ROS into signaling pathways averts the oxidative damage and induces tolerance to an individual or, may be, to a set of stresses.

### 3.1. Photosynthetic Apparatus (Chloroplast)

**Figure 3:**
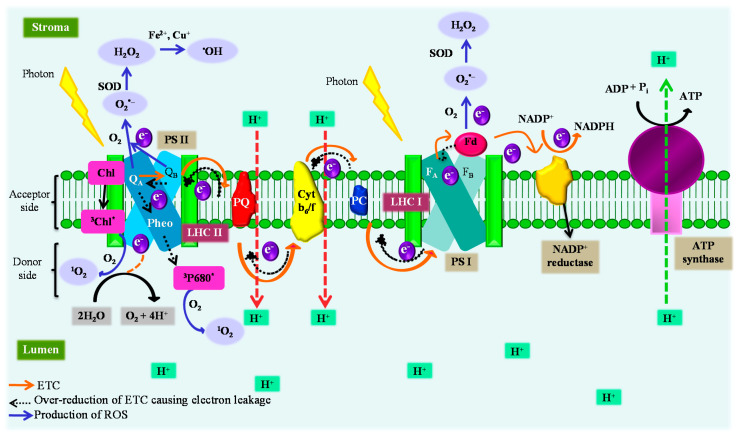
Photosynthetic electron transport chain under abiotic stress gets over-reduced and triggers the production of ROS. Photons striking at light-harvesting complex I and II (LHC I and II) result in electron (e^−^) generation and hydrogen or proton (H^+^) gradient, which initiates the electron transport chain (ETC) at photosystem (PS) I and II (through photolysis of H_2_O) and production of NADPH and ATP by NADPH reductase and ATP synthase, respectively. However, the excess illumination of photons at LHC II converts the chlorophyll (Chl) molecule into an excited triplet form (^3^Chl*), which reduces O_2_ to ^1^O_2_. The reduced activity of Calvin’s cycle due to low CO_2_, leads to the over-reduction of ETC causing electron leakage. The electron moves in reverse from PS I to II and at PS II from Q_B_ to Q_A_ and then to the pheophytin forming an excited triplet chlorophyll (^3^P680), which reduces O_2_ to ^1^O_2_. Over-reduction of Q_B_ and Q_A_ also directly reduces O_2_ to O_2_**^•^**^–^. At PS I, the over-reduction of ETC prompts electron leakage from ferredoxin (Fd) to O_2_ forming O_2_**^•^**^–^ via Mehler’s reaction. The O_2_**^•^**^–^ generated is dis-mutated either spontaneously or by the action of superoxide dismutase (SOD) to H_2_O_2_, which in the presence of reduced redox metals (Fe^2+^, Cu^+^) changed to a highly toxic ^•^OH.

The photosynthetic apparatus (chloroplast) is an extremely important plant cell organelle that generates energy to drive life on earth. The chloroplast is susceptible to hostile conditions and a prime site for ROS generation (Figure 3). ROS produced within the chloroplast reduces the photosynthetic efficiency leading to dwindling growth and productivity. Exploring molecular processes affecting the photosynthetic activity and excess ROS generation may prevent deleterious effects. Adverse environmental conditions reduce stomatal conductance, decrease CO_2_ assimilation, and/or result in the formation of excited triplet chlorophyll (^3^Chl*) that disturbs photosynthetic ETC, induces overproduction of ROS, and prompts photo-oxidation [84]. ROS are generated at the reaction center of PS I and II mainly due to the presence of excess high energy-intermediates, reductants, and O_2_ [85,86]. Upon illumination, light-harvesting complexes (LHC) absorb energy (photon) and produce an excited singlet chlorophyll (^1^Chl*), which is a long-lived molecule and participates in the conversion of excitation energy into electrochemical energy via charge separation. In the presence of excess light, energy absorbed by LHC at the acceptor side of PS II exceeds over its utilization threshold limit and results in the formation of ^3^Chl* [87]. ^3^Chl* reacts with O_2_ leading to the generation of highly oxidizing ^1^O_2_. Apart from the excess light, other stresses such as drought induce disequilibrium between the light capture and its utilization, resulting in the production of ^1^O_2_ [88]. Abiotic stresses limit the availability of CO_2_ to Calvin’s cycle due to the reduced stomatal conductance, causing an over-reduction of plastoquinone Q_A_ and Q_B_ (photosynthetic ETC component of PS II) that hinders the charge separation between P680 (chlorophyll molecules present at PS II) and pheophytin. The phenomenon triggers the formation of triplet chlorophyll (^3^P680) at the PS II reaction center, which ultimately leads to the production of ^1^O_2_ [89]. Due to the low concentration of CO_2_ (final electron acceptor) under abiotic stress conditions, the decreased availability of NADP^+^ prompts excessive electron leakage from the photosynthetic electron transport and reduces O_2_ at the acceptor side of PS I via ferredoxin into O_2_**^•^**^–^ known as Mehler’s reaction [89,90]. The over-reduction (overloading) of photosynthetic ETC causes electron leakage from plastoquinone Q_A_ and Q_B_ to O_2_ resulting in the generation of O_2_**^•^**^–^ at the reaction center of PS II [91]. An increased thylakoid membrane electron leakage to O_2_ under drought has been reported in sunflower by Sgherri et al. [92]. Excitation of O_2_ by highly energized chlorophyll pigments also results in the formation of O_2_**^•^**^–^ [8]. The O_2_**^•^**^–^ is then converted into a stable H_2_O_2_ either spontaneously or by dismutation via the action of thylakoid membrane-bounded/stromal membrane Cu/Zn-SOD [86,93]. The H_2_O_2_ generated is a potential photo-inhibitor that causes oxidation of cysteine (Cys) or methionine (Met) residues [89] and thiol modulated enzymes of Calvin’s cycle inhibiting CO_2_ fixation by 50% even at a concentration of 10 µM [93]. H_2_O_2_ has also been reported to mediate the signaling pathway, hence modulation of H_2_O_2_ into the signaling can avert oxidative damages. H_2_O_2_ undergoes further transformation leading to the formation of highly reactive and most toxic ^•^OH through the Fenton reaction mediated by redox metals (Fe^2+^ or Cu^+^) [87]. However, quenching excess redox metals from chloroplast can prevent the Fenton reaction and production of ^•^OH that can bridge associated damages. Therefore, studies need to be carried out to elucidate mechanisms for intrinsically sequestering excess redox metals.

ROS produced in the chloroplast results in photo-oxidative stress leading to lipid peroxidation, damage to the membrane protein that affects the PS II reaction center, and ultimately cell death [94,95]. For instance, herbicides such as bentazon, paraquat, and 3-acetyl-5-isopropyltetramic acid inhibit photosynthesis and trigger ROS generation by competing with the D1 binding site of plastoquinone and blocking photosynthetic ETC from PS II [96] and/or by inhibiting the ultimate electron acceptor of PS I, i.e., NADP^+^ and accepting an electron from PS I, which finally actuates the production of O_2_**^•^**^–^, H_2_O_2_, and ^•^OH [85]. Analogously, the availability of NADP^+^ to electrons reduces the under chilling stress that disrupts ETC and elicits ROS generation [42]. The chilling stress also induces overexcitation of the thylakoid membrane, which causes photo-inhibition and impairs the functioning of the photosynthetic machinery [42,97]. Yamane et al. [98] and Shu et al. [99] have reported damage to the chloroplast ultrastructure, i.e., destruction of chloroplast membrane, swelling of thylakoid, and aberrations in the thylakoid membrane, which is attributed to the production of ROS such as H_2_O_2_ and O_2_**^•^**^–^ under salinity stress. Pandey et al. [68] have reported increased production of O_2_**^•^**^–^, H_2_O_2_, and ^•^OH in the pea plant chloroplast exposed to Cr (VI). Similarly, the inhibition of PS II, ATP synthetase, enzymes of Calvin’s cycle, disruption of photosynthetic ETC, and ROS production in the presence of metals such as nickel (Ni), Cd, Cu, Zn, and Cr has been reported by Dietz et al. [100] and Shahzad et al. [29]. Shakirova et al. [84] have observed the oxidative stress in wheat exposed to Cd resulting in the production of MDA and increased electrolyte leakage.

### 3.2. Peroxisomes

**Figure 4:**
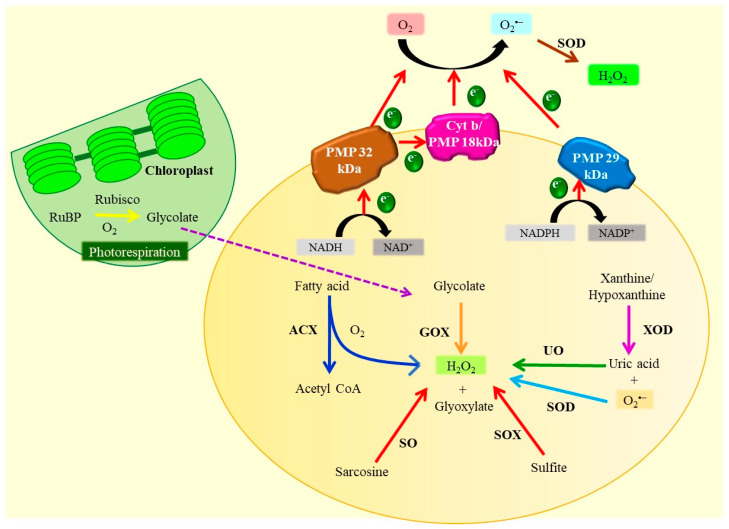
Different pathways for ROS production in peroxisomes under abiotic stress. ROS in the peroxisomes matrix is generated via the action of different enzymes. Glycolate produced in the chloroplast during photorespiration moves to peroxisomes where the action of glycolate peroxidase (GOX) generates glyoxylate and H_2_O_2_. The fatty acid undergoes β-oxidation in the presence of enzyme acyl-CoA oxidase (ACX) leading to the production of acetyl CoA and H_2_O_2_ [104]. Xanthine oxidase (XOD) catalyzes xanthine and/or hypoxanthine into the uric acid and O_2_**^•^**^–^. The uric acid gets catalyzed by urate oxidase (UO) resulting in the production of H_2_O_2_ [105]. Other compounds such as sarcosine and sulfite undergo oxidation in the presence of enzymes sarcosine oxidase (SOX) and sulfite oxidase (SO) in peroxisomes and generate H_2_O_2_ [101]. NAD(P)H dependent small ETC consisting of three peroxisome membrane polypeptides (PMPs)—32, 18, and 29kDa generate ROS through electron leakage. NADH releases an electron to PMP 32kDa (NADH ferricyanide reductase) and forms NAD^+^ (oxidized nicotinamide adenine dinucleotide), the electron either reduces O_2_ to O_2_**^•^**^–^ in cytosol or moves to cytochrome b (Cyt b/PMP 18kDa) where it reduces O_2_ to O_2_**^•^**^–^ in the cytosol. At PMP 29kDa, NADPH regenerates NADP^+^ releasing electron which reduces O_2_ to O_2_**^•^**^–^ in the cytosol. O_2_**^•^**^–^ forms dis-mutate either spontaneously or in the presence of superoxide dismutase (SOD) into H_2_O_2_.

Peroxisomes are another major site for intracellular H_2_O_2_ production [101]. They also operate several important cellular functions, including high oxidative metabolic pathways in most of the eukaryotic cells [95,102] (Figure 4). Apart from H_2_O_2_, O_2_**^•^**^–^ are also produced in the matrix and/or at the membrane of peroxisomes and are released into the cytosol [103]. The processes such as photorespiration, fatty acid β-oxidation mediated by acyl CoA oxidase (ACX), and the activity of enzymes such as flavin oxidase, urate oxidase (UO), xanthine oxidase (XOD), etc. in peroxisomes partake in ROS generation [104,105]. Under abiotic stress such as flooding, drought, salinity, high irradiance, heavy metals, xenobiotic compounds, high temperature, or chilling, the process of photorespiration initiates in the chloroplast due to the limited availability of CO_2_ and increased solubility of O_2_ that competitively accelerate the oxygenation of ribulose-1,5-biphosphate [106,107] to produce glycolate, which then gets exported to peroxisomes where glycolate oxidase (GOX) oxidizes it, generating H_2_O_2_ [90,95].

Yamane et al. [98] have reported that salinity stress enhances the photorespiration and H_2_O_2_ level in peroxisomes. The increased lipid peroxidation and reduced activity of the ascorbic acid (AsA) and glutathione (GSH) in tomato plants subjected to salt stress have been reported by Mittova et al. [108]. The salt stress-induced oxidative damage probably arises from the production of ROS by the activity of peroxisomal GOX [106]. During drought conditions, photorespiration is estimated to contribute to >70% of H_2_O_2_ generation [106]. Further, β-oxidation of fatty acids, activities of enzymes such as flavin oxidases, XOD, UO, and disproportionation of O_2_**^•^**^–^ trigger the production of H_2_O_2_ in peroxisomes [102,103,105]. Under abiotic stress characterized by prolonged darkness, chloroplasts release fatty acids which subsequently get metabolized by the peroxisomal β-oxidation [109]. Ortega-Galisteo et al. [103] have reported that the Cd and 2,4-dichlorophenoxyacetic acid (2,4-D) induced the production of H_2_O_2_ in pea leaves. Cd increases the H_2_O_2_ level due to the increased activity of GOX and reduces the CAT activity, whereas 2,4-D elevates ACX (β-oxidation of fatty acids) and XOD activities. The number of peroxisomes in plant cells also proliferate in the presence of abiotic stress including xenobiotic compounds, salinity, O_3_, heavy metals, salinity, and high light [102]. Another important ROS, O_2_**^•^**^–^ is produced in peroxisomes on the action of salinity, Cd, herbicides, and other xenobiotics [110]. Peroxisomal O_2_**^•^**^–^ is generated via two different mechanisms. The first mechanism involves peroxisome membrane-localized NADH dependent small ETC comprising peroxisomes membrane polypeptide (PMP)-NADH: Ferricyanide reductase and cytochrome (Cyt) b of molecular masses 32 and 18kDa, respectively. NADH dependent ETC oxidizes NADH and Cyt b as well as reduces O_2_ to O_2_**^•^**^–^ which is released into the cytosol [8,95]. In addition to PMP 32kDa and PMP 18kDa, another PMP of about 29kDa molecular mass generates O_2_**^•^**^–^ using NADPH as an electron donor and reduces Cyt c [95,102]. The second mechanism includes the oxidation of xanthine and hypoxanthine to uric acid with a simultaneous production of O_2_**^•^**^–^ mediated by XOD present in the peroxisomal matrix [8,95]. *A. thaliana* seedlings exposed to Cd stress overproduce O_2_**^•^**^–^ in peroxisomes [111]. Similarly, pea plants exposed to Cd stress exhibit an increased number of peroxisomes, O_2_**^•^**^–^ and H_2_O_2_ overproduction, and alteration in some endogenous proteins [112,113].

### 3.3. Mitochondria

**Figure 5:**
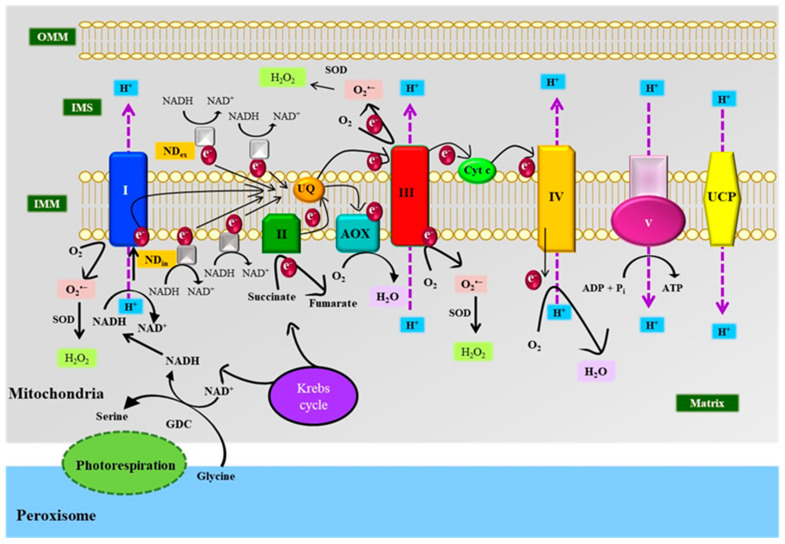
Mitochondrial electron transport chain (mtETC) mediated ROS production and alternative pathway under abiotic stress. The process, such as photorespiration and Krebs cycle, results in the generation of NADH and/or succinate which enters mtETC at complex I or complex II, respectively. At complex I, NADH converts into NAD^+^ and H^+^ with the generation of an electron. At complex II, succinate is changed to fumarate with the electron generation. An electron from both complex I and II get transferred to UQ from where they move to complex III and then to complex IV via Cyt c. The electron at complex I and III reduces O_2_ to generate ROS (O_2_**^•^**^–^ and H_2_O_2_), whereas, at complex IV, O_2_ oxidized to H_2_O. H^+^ generates at complex I, III, and IV pumped to IMS and then moves to complex V or ATP synthase to form ATP from ADP. The mtETC also comprises an alternative pathway consisting of two each NDex and NDin with AOX and UCP which limit ROS generation. NDex and NDin function in stress conditions and transfer electrons to UQ. The AOX present between UQ and complex III accepts an electron from UQ and reduces O_2_ to H_2_O, thus terminating the electron transport to complex III. OMM: Outer mitochondrial membrane; IMS: Inter-mitochondrial space; IMM: Inner mitochondrial membrane; e^−^: electron; UQ: Ubiquinone; I–V: Complex I–V; Cyt c: Cytochrome c; NDex and NDin: NAD(P)H dehydrogenase on the exterior and interior side of IMM, respectively; AOX: Alternative oxidase; UCP: Uncoupling protein.

Mitochondria are the other potential site for the production of O_2_**^•^**^–^, H_2_O_2_, and ^•^OH in plants (Figure 5). Mitochondrial ETC (mtETC) and photorespiration favor ROS formation under abiotic stress. The mtETC or respiratory ETC operates in the inner membrane of mitochondria through two pathways, i.e., cytochrome oxidase (COX) with the ATP synthesis and alternative oxidase (AOX)-cyanide insensitive pathway without the ATP synthesis [114]. The mtETC comprises four oxido-reductase complexes I-IV (complex I-NADH dehydrogenase; complex II-succinate dehydrogenase; complex III-Cyt c reductase; complex IV-COX), two interior alternatives (NDin), and two exterior alternatives (NDex) NAD(P)H dehydrogenases (rotenone), one ATP synthase (complex V), mobile ubiquinone (UQ), mobile Cyt c, AOX, and uncoupling proteins (UCPs) [115]. A constraint on respiration during stress causes an over-reduction of mtETC that stimulates electron leakage to O_2_ and ROS production [94,116]. The input of electron to mtETC when it exceeds more than its ability to utilize, over-reduces the UQ pool accelerating ROS generation [117]. Complex I and III of mtETC partake in ROS generation [95], whereas alternative NDs, AOX, and UCP are known to reduce the ROS production under stress [114,118]. O_2_**^•^**^–^ gets produced through the reduction of O_2_ at the flavoprotein region and iron-sulfur (Fe-S) center of NADH dehydrogenase and/or by Cyt c reductase due to the reduction of UQ, which favors leakage of an electron to O_2_ by generating highly reducing ubisemiquinone radicals [95]. Under drought and/or salinity stress, the over-reduction of the UQ pool in mitochondria due to the perturbation of ETC favors the production of ROS [94,95,98,119]. Hu et al. [120] have obtained similar results under chilling stress. Exposure to stress results in the over-reduction of mtETC and electron leakage to O_2_ forming O_2_**^•^**^–^. Concomitantly, heat stress-induced hyperpolarization of the mitochondrial inner membrane of winter wheat cells due to the high potential gradient accelerates the over-reduction of the respiratory electron chain and actuates the production of ROS [121]. Complex II (succinate dehydrogenase) indirectly contributes to the ROS load in mitochondria by reversing the electron flow towards complex I due to the dearth of NAD^+^ (oxidized nicotinamide diamine dinucleotide)–linked substrate [122]. This reverse electron flow from complex II to I is regulated by ATP hydrolysis [109]. O_2_**^•^**^–^ is the major ROS produced in the mitochondria, which disproportionates into H_2_O_2_ by the activity of Mn-SOD and APX [8,89]. H_2_O_2_ formed in the presence of reduced Fe^2+^ or Cu^+^ yields a highly toxic ROS radical ^•^OH via the Fenton reaction [116]. Photorespiration occurring in peroxisomes under stress conditions produces glycine which enters the mitochondria where it gets converted to serine and reduces NAD^+^ to NADP by the action of glycine dehydrogenase complex (GDC) in the mitochondrial matrix [115]. In an excess light condition, GDC is probably the main substrate that produces NADP and donates an electron to complex I which initiates ETC [115] and may induce O_2_**^•^**^–^ formation. The ROS generated in the mitochondria under stress affect its structure [123] and function, sometimes even leading to programmed cell death (PCD). Yamane et al. [98] have suggested that H_2_O_2_ generated under salinity stress is probably responsible for the degradation of mitochondrial cristae. Overproduction of ROS in the mitochondria leads to lipid peroxidation and PCD. This results in an alteration in the membrane potential, prompting the release of intermembrane space localized Cyt c to the cytosol [107,124,125]. The translocation of Cyt c from the mitochondria to cytosol has been observed in cucumber under heat stress [126]. Gao et al. [125] have reported the activation of caspase-like protease, DNA laddering, nucleus fragmentation, and PCD in *A. thaliana* due to the mitochondrial transmembrane potential loss and ROS formation after exposure to excess UV radiation.

### 3.4. Plasma Membrane, Cell Wall, and Apoplast

**Figure 6:**
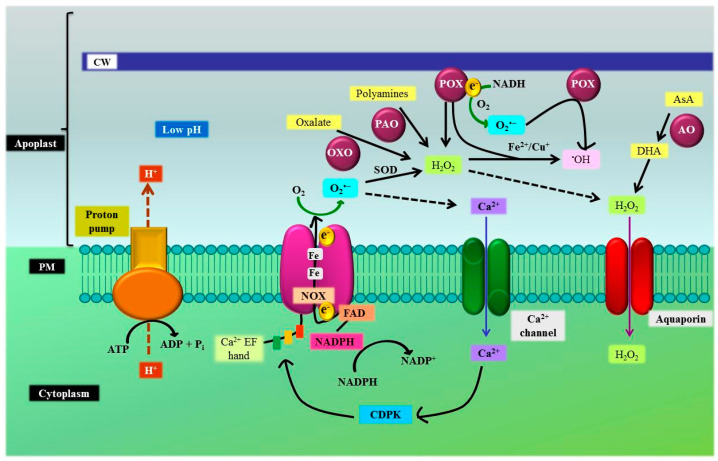
ROS production by the plasma membrane, apoplast, and cell wall under abiotic stress. The plasma membrane (PM) localized NADPH oxidase consists of two cytoplasmic binding sites: 1) Flavin adenine dinucleotide (FAD) and nicotinamide adenine dinucleotide phosphate (NADPH) and 2) Ca^2+^ binding EF-hand motifs. The NADPH oxidase transfer electron from the cytosolic NADPH to the apoplast via cytochrome (Fe) present in the channel is formed by NADPH oxidase transmembrane domains and reduces O_2_ to O_2_**^•^**^–^. In the apoplast, O_2_**^•^**^–^ either spontaneously (due to low pH maintained through the proton pump) or by the action of SOD disproportionates into H_2_O_2_. O_2_**^•^**^–^ induces the Ca^2+^ influx through the Ca^2+^ channel which moves to the Ca^2+^ binding EF-hand motif of NADPH oxidase via the calcium-dependent protein kinase (CDPK) and activates the NADPH oxidase leading to ROS production. Other enzymes such as cell wall (CW) bound peroxidases (POX) and apoplast localized amine oxidases (AO), polyamine oxidases (PAO), and oxalate oxidases (OXO) in the presence of specific substrates result in ROS generation. POX in the presence of NADP reduces O_2_ to O_2_**^•^**^–^ which dis-mutates to H_2_O_2_. Similarly, AO breaks down AsA to dehydroascorbate (DHA), which in turn generates H_2_O_2_. OXO and PAO partake in H_2_O_2_ formation in the presence of oxalate and polyamine, respectively. H_2_O_2_ is converted into ^•^OH either through the Fenton reaction in the presence of redox metals or by the action of POX. H_2_O_2_ produced in the apoplast also moves to the cytoplasm through aquaporins and participates in signaling.

The plasma membrane and apoplast envelope the cell organelles and maintain cell activity, fluidity, rigidity, ion transport, as well as secure its integrity [83,127]. Plasma membrane-localized NADPH oxidases are major ubiquitous enzymes that catalyze reactions generating ROS [82,83]. NADPH oxidases mediate the transfer of an electron from cytosolic NADPH to O_2_ which results in the production of O_2_**^•^**^–^ in the apoplast [128] that undergoes dismutation either spontaneously or by the action of antioxidant enzyme SOD, yielding H_2_O_2_ [95,129] (Figure 6). During oxygen depriving stress conditions (hypoxia), the plasma membrane located NADPH oxidase partakes in the production of H_2_O_2_ in the apoplastic space [130]. The apoplast produces extracellular ROS such as H_2_O_2_ under sub-optimal conditions. The pathway for ROS production operates under cell wall-associated enzymes including pH dependent extracellular POXs, quinine reductase, lipoxygenases, amine oxidases (AO), polyamine oxidases (PAO), and germin-like oxalate oxidases (OXOs) [83,129,131] (Figure 6). H_2_O_2_ is constantly generated in the apoplast on the combined action of abscisic acid and stress signals [132]. Voothuluru and Sharp [133] have reported an increase in apoplastic H_2_O_2_ content in the primary root of maize, experiencing a water-deficient condition which is mediated by the activity of the OXO enzyme. Lin and Kao [134] have also recorded a reduced root growth of rice seedlings grown under salinity stress impacted by increased activity of cell-wall POX, NADH peroxidase, and DAO which promote the accumulation of H_2_O_2_ in the cell wall. Other abiotic stress such as the presence of ground-level O_3_ induces oxidative burst in a plant cell by actuating the production and accumulation of H_2_O_2_ and O_2_**^•^**^–^ in the apoplast which inflicts necrosis and cell death [135]. The production of H_2_O_2_ by the cell wall-associated POX in *Arabidopsis* under the K^+^ deficient condition has been reported [136]. Stress conditions such as salinity/osmotic stress activate NADPH oxidases, apoplastic DAO, and PAO enzymes which promote the production of ROS [137]. PAO catabolizes polyamines such as spermidine and produces/releases H_2_O_2_ as a byproduct in the apoplast under high salinity stress [138].

## 4. Biomolecules Targeted by ROS and Oxidative Damage

**Figure 7:**
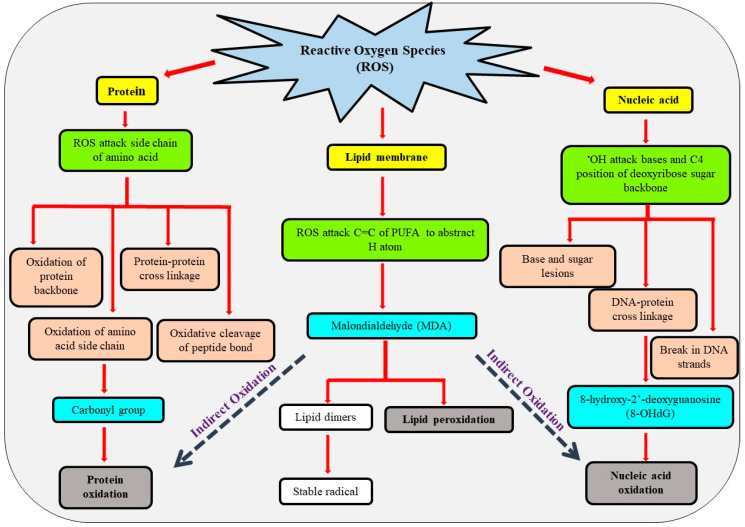
Reactive oxygen species attack biomolecules (proteins, membrane lipids, and nucleic acids) at different sites leading to oxidation that alters their structural and functional activities. Oxidation of biomolecules results in the formation of carbonyl group, malondialdehyde, and 8-hydroxy-2′-deoxyguanosine, which are considered as a best marker of protein, lipid, and nucleic acid oxidation, respectively.

ROS overproduction leads to oxidative burst and damage to biomolecules under adverse environmental conditions (Figure 7). The damaged biomolecules comprise the product of protein oxidation, inactivation of enzymes, lipid peroxidation, increase membrane fluidity, chlorophyll degradation, nucleic acid damage, and commencement of the apoptosis pathway and PCD in severe conditions [9,80]. These damages affect the growth, development, and ultimately plant survival. The extent of damage to biomolecules depends on various factors including the concentration of particular biomolecule(s), location of the target biomolecule(s) in relation to the site of ROS generation, the rate constant for the reaction between target biomolecule(s) and ROS, the occurrence of secondary damaging incidents and ROS scavenging or detoxifying repair system [139].

### 4.1. Lipid Membrane

The oxidative burst in a cell under stress conditions damages the lipid membrane. Lipid peroxidation reactions involve lipoxygenase activity, ^1^O_2_ generation, and radical catalyzed mechanism, which differ quantitatively between underground and aboveground tissues depending on the type of ROS [140]. ROS targets unsaturated C-C double bond polyunsaturated fatty acids (PUFA), e.g., arachidonic acid, linolenic acid, and linoleic acid. The ^•^OH radical attacks on the methylene group of fatty acid and abstracts the hydrogen (H) atom forming carbon-center lipid radical [141]. ROS also breaks the ester linkage between glycerol and fatty acids, disintegrating membrane phospholipids [89,95]. The ROS radical, ^•^OH initiates the cyclic reaction resulting in peroxidation of PUFA [95]. The process of lipid peroxidation involves three stages: Initiation, propagation, and termination (cleavage) [141]. Initiation involves the production of ROS by the reduction of O_2_. The ROS generated trigger a cascade of reactions leading to the formation of lipid radicals (lipid peroxyl radicals, hydroperoxides, etc.) and MDA conforming the second stage, i.e., propagation. Finally, lipid radicals end up as the formation of lipid dimmers [95]. Lipid peroxidation perpetrates membrane destabilization with regards to permeability, electrolyte leakage, deactivation of enzymes and receptors as well as enhances the oxidation of nucleic acids and proteins [89,95,141]. Lipid radicals generated during lipid oxidation undergo enzymatic or non-enzymatic degradation and yield compounds such as reactive carbonyl species (RCS) [142]. These RCS selectively react with proteins via the lipoxidation reaction and result in the loss of functional activities of proteins. The alleviating degradation of lipid radicals by their direct elimination from a cell can prevent the lipoxidation reaction and further oxidative damage to the cell. For example, Gram-positive bacterium *Deinococcus radiodurans* possesses the ability to reduce ROS induced injury to Fe-S proteins by eliminating the cellular iron outside the cytosol [140]. Studying the underlying mechanisms for sequestration of susceptible or damaging molecules can provide avenues to enhance tolerance in plant cells against oxidative damage.

Abiotic stresses such as salinity [143], temperature [144], metals and metalloids [145,146], drought [147,148], xenobiotic compounds including pesticides [149], ground-level O_3_ [150], and UV radiation [151] converge oxidative stress to accelerate cellular and organelle lipid peroxidation. ROS in roots of rice seedlings exposed to excess aluminum exhibit lipid peroxidation, as well as DNA damage [152]. Arsenic (As) stress also prompts H_2_O_2_ accumulation, lipid peroxidation, and electrolyte leakage in common bean seedlings [153]. The lipid peroxidation product, MDA, indicates the degree of oxidative damage in the cell and hence a marker for the degree of the damage [89,95]. Martinez et al. [154] have reported an over-accumulation of H_2_O_2_ followed by a high MDA content and lipid peroxidation in tomato plants exposed to salinity, heat, and combined stresses. Kumari et al. [150] have also demonstrated a significant increase in lipid peroxidation/electrolyte leakage and reduction in the chlorophyll content and photosynthetic rate in *Solanum tuberosum* L. cv. Kufri chandramukhi grown under ambient CO_2_ and elevated O_3_. Membrane lipid peroxidation in *Phalaenopsis* due to the exposure to an elevated temperature induces the loss of membrane integrity and K^+^ leakage [144].

### 4.2. Proteins

Proteins play a crucial role in mediating tolerance to abiotic stress by adjusting the physiological characters of plants [155]. Protein aggregation or change in conformation affects their enzymatic, binding, and other functional activities [141,156]. Proteins are more susceptible to oxidation than other biological molecules due to their abundance in the living system and high-rate constants for the reaction [140]. Both radical and non-radical oxidants cause protein oxidation either directly or indirectly. Some ROS cause little and selective damage to certain residues, while others such as ^•^OH induce widespread and non-selective (non-specific) damages [140]. The protein backbone attacked by non-radical oxidants results in limited damage due to the slow oxidation rate. However, the extensive or widespread damage to the protein backbone is induced by radicals that react rapidly primarily through the abstraction of the H atom at the α-carbon site resulting in the formation of stabilized carbon-center radicals [140]. The direct oxidation by ROS involves both, oxidation of side chains of amino acid specifically those containing sulfur (S) and thiol groups (e.g., oxidation of Cys and Met residue by ^1^O_2_ and ^•^OH) and degradation of peptide backbone resulting in carbonylation, nitrosylation, disulfide bond formation, and glutathionylation, which alters the protein activity [95]. On the contrary, indirect oxidation is mediated via products formed during lipid peroxidation [95,157]. The oxidation of protein enhances their susceptibility towards proteolytic digestion [158] by getting prepared for ubiquitination-mediated degradation by the proteasome [95]. Protein oxidation by ROS is either irreversible or reversible. ROS such as O_2_**^•^**^–^ can irreversibly damage enzymes that contain the Fe-S center [95]. The irreversible damage to protein such as carbonylation, protein-protein cross-linking, etc. causes functional loss. On the other hand, reversible changes such as glutathionylation and *S*-nitrosylation can mediate the redox regulation [141]. Carbonylation of the protein is irreversible and an unrepairable damage mediated by the oxidative cleavage of proteins and is considered as the best marker for estimation of oxidative damage under stress [158]. Oxidation of heat shock proteins and late embryogenesis abundant (LEA) proteins reduces the capacity to maintain protein functioning in dehydrated seeds [159]. Karuppanapandian and Kim [160] has noted a significant increase in the carbonylated protein in cobalt-stressed *B. juncea* leaves. Carbonylated proteins occur in plant cell organelles including chloroplast, mitochondria, nucleus, cytosol, and peroxisomes [141]. Under drought conditions, the protein carbonyl level elevated by several folds has been detected in the mitochondria as compared to the chloroplast and peroxisomes in leaves of the wheat plant [161]. An increase in protein oxidation has been demonstrated in cashew plants subjected to salinity stress [162]. Exposure of *A. thaliana* and *Glycine max* to excessive CO_2_ also induces protein carbonylation [163]. Oxidative damage to proteins under chilling, paraquat, and O_3_ stress leads to functional loss, which has been demonstrated in several studies [164,165,166].

### 4.3. Nucleic Acid

Nucleic acids undergo oxidation on the ROS attack that affect protein synthesis and may lead to mutation [89,167]. The DNA present in the chloroplast and mitochondria are more susceptible to oxidation than the DNA present in the nucleus due to their proximity to the ROS production site and lack of protective histones and associative proteins [95]. ROS imperil oxidation of nucleic acid that includes the oxidation of sugar residue, alteration of nucleotide bases (insertion or deletion), and the abstraction of nucleotide break in the DNA strand, cross-linking the DNA and protein [95]. Intersomal nDNA fragmentation has also been reported in the sensitive genotype of wheat with PCD in leaves under drought [148]. The ROS subtract H-atom from the C4 position of deoxyribose sugar backbone forming the deoxyribose radical, further causes a break in DNA strand [168]. Among all ROS, the ^•^OH radical has been reported to cause maximum damage to DNA due to its ability to react with purines/pyrimidine bases, and even deoxyribose sugar [169]. Apart from ^•^OH, ^1^O_2_ reacts only with guanine, while O_2_^•–^ and H_2_O_2_ do not react with any purine or pyrimidine bases [169]. The ^•^OH radical attacks the double bond of purines and pyrimidine bases [170] developing DNA lesions and forming 8-hydroquinine and some other less common products such as hydroxyl methyl urea, thymine glycol, etc. [95]. The cross-linking between DNA and protein is also facilitated by the ^•^OH radical by reacting either with the DNA or associated proteins. The repairing of this cross-linkage is a difficult task, and if not repaired before replication or transcription, can cause a lethal effect on the plant cell [95]. In addition to the direct oxidation of DNA, the lipid radicals obtained from lipid peroxidation prompt an indirect DNA oxidation [171]. MDA, a major product of lipid peroxidation reacts with guanine (G) residues in the DNA to form M_1_G, i.e., pyrimidopurinone adduct [172]. RNAs are also susceptible to the ROS attack [173]. Oxidation of RNA results in the formation of 8-oxo-7,8-dihydroguanosine (8-OHG), which is used as a marker for the determination of the intensity of RNA oxidation. The Cd-induced oxidation of RNA in soybean seedling [174] and degradation of mRNA during water deficit stress (desiccation) in *Lindernia subracemosa* [175] have been documented. ROS affect the DNA replication and transcription that may abnormally affect the protein synthesis, membrane stability, as well as signal transduction pathways in a cell, reducing metabolic efficiency, genetic instability, and compromising cell homeostasis [169]. The accumulation of radicals formed due to the oxidization of the biomolecule shows the potential to oxidize other biomolecules, which may elevate oxidative damage in plant cells and result in PCD under a severe condition. To avoid extensive damage, the continuous elimination or repairing of damaged biomolecules is necessary. Fortifying plants’ intrinsic mechanisms to remove or repair damaged biomolecules may induce the resistance towards stress and prevent productivity losses.

## 5. Antioxidants: Oxidative Stress Defense Mechanism

ROS at a low or moderate concentration act as a secondary messenger and participate in a signaling cascade within the cell that elicit a response to tide over stress situations [8,89]. Ironically in stress conditions, ROS are generated in high concentrations that become toxic and are responsible for PCD [116]. The activity of ROS in the plant cell (regulative, damaging, or signaling) depends on the equilibrium between their production and detoxification system [176]. Enzymatic and non-enzymatic antioxidants of plants act as ROS detoxifying machinery, which limit their concentration and maintain their steady-state level inside cellular compartments [154,177]. Enhancing the antioxidant level of plant cells either endogenously through genetic engineering or by an exogenous application can strengthen the defense system of the plant and rescue them from the debt of environmental stress.

### 5.1. Enzymatic Antioxidants

An enzymatic antioxidant such as SOD, CAT, APX, POX, monodehydroascorbate reductase (MDHAR), dehydroascorbate reductase (DHAR), glutathione *S*-transferase (GST), glutathione peroxidase (GPX), AOXs, peroxiredoxin (Prx), and thioredoxin (Trx) alleviates the ROS level by breaking them down and removing them from the system through various steps including conversion of ROS to H_2_O_2_ and then to the H_2_O molecule in the presence of metallic co-factors [178]. SOD (EC: 1.15.1.1) are metalloenzymes that are found in three isoforms viz. Cu, Zn-SOD localized in the cytosol, chloroplast, peroxisomes, nuclei, mitochondria, and apoplast; Fe-SOD in chloroplast, peroxisomes, and mitochondria; and Mn-SOD in peroxisomes, mitochondria, and vascular tissues [116,179,180]. SODs provide an initial or first line of defense against toxic ROS [116]. They catalyze the disproportionation of O_2_**^•^**^–^ free radicals by reducing one radical into H_2_O_2_ and oxidizing another into O_2_ thereby eliminating the risk of production of more toxic free radical ^•^OH [116]. CAT (EC: 1.11.1.6), APX (EC: 1.11.1.11), and GR (EC: 1.6.4.2) catalyze the decomposition of H_2_O_2_ antioxidant into H_2_O and O_2_ [181,182]. CAT and APX are metalloenzymes localized in peroxisomes and mitochondria [182]. Apart from these, APX is also found in the cytosol, chloroplast, microbodies, and peroxisomes/glyoxysomes [86,183] and participates in the AsA-GSH (ascorbate-glutathione) pathway as a key enzyme [184,185]. The enzyme requires AsA as a reducing substrate for its stability and proper functioning [186]. AsA-GSH or Foyer-Halliwell-Asada pathway [184], comprising enzymatic (APX, MDHAR, DHAR, GPX) and non-enzymatic (AsA, GSH) antioxidant components, operates in chloroplast, plastids, mitochondria, and peroxisomes [185] to combat the overproduction of H_2_O_2_ [187].GR is a flavoprotein oxido-reductase that is localized in the chloroplast (where it displays 70–80% of the activity) [116,188], mitochondria, cytosol, peroxisomes, and in non-photosynthetic tissues and organelles [189]. Its activity is dependent on the combined action of pH and concentration of NADPH and glutathione disulfide (GSSG) at the site of action [188]. GR catalyzes the conversion of oxidized GSH (GSSG) into the reduced form-GSH using NADPH as an electron donor [116] and maintains a balance between the GSH/GSSG ratio necessary for the detoxification of H_2_O_2_ [190]. Prxs are thiol peroxide enzymes that detoxify peroxidase substrates such as H_2_O_2_ and alkyl hydroperoxide and reduce oxidative damage [191,192]. Prx participates in ROS dependent signaling by modulating the concentration of H_2_O_2_, processing alkyl hydroperoxide, switching to chaperone function, etc. [192]. Prxs contain one or two catalytic Cys in a conserved sequence and are classified into four groups, (1) 1-Cys Prx, (2) 2-Cys Prx, (3) YLR109-related Prx, or type II Prx, and (4) bacterioferritin-comigratory protein or Prx Q [191,193]. Trxs are small thiol-disulfide regulatory proteins (around 14kDa) that reduce the disulfide bond and participate in ROS regulation [192]. Trxs contain a pair of cysteinyl residues in a highly conserved amino acid motif WC[G/P]PC, which are involved in the catalytic activity of the enzyme [194].

### 5.2. Non-Enzymatic Antioxidants

Non-enzymatic antioxidants detoxify ROS by interrupting a free-radical chain reaction [179]. The non-enzymatic compounds such as AsA, GSH, compatible solutes, phenolics, α-tocopherol, carotenoids, flavonoids, and even proline counteract the uncontrolled cascade of ROS produced during stress [123,137,195]. GSH is a ubiquitous thiol tripeptide that participates in the degradation of H_2_O_2_ in a reaction catalyzed by GPX [196]. It takes part in the AsA-GSH pathway as a reductant for DHAR and aids in the scavenging of H_2_O_2_ [187,196] and/or degradation of H_2_O_2_ and lipid peroxides by forming a conjugate through a reaction catalyzed by the GPX and GST, respectively [196]. AsA or vitamin C participates in the AsA-GSH pathway as an electron donor for APX [168] and is a co-factor of POXs [197]. AsA helps in the regeneration of tocopherol and xanthophyll production that partakes in quenching of the excitation energy [173]. Carotenoids are a light-harvesting pigment [198,199] that alleviates high light illumination induced oxidative stress by quenching excessive energy as heat dissipation [198,199]. Carotenoids also avert the over-excitation of PS II in the thylakoid membrane by efficiently scavenging ^1^Chl*, ^3^Chl*, and ^1^O_2_ [199].

The gamma-aminobutyric acid (GABA) is a ubiquitous non-protein amino acid that accumulates in plant cells under stress conditions and provides tolerance by scavenging free radicals and regulating the enzyme activity [36,200]. GABA acts as an osmolyte or encourages the production of other osmolytes such as proline under conditions such as the drought that aids in osmotic adjustment for acclimatization during stress [36]. GABA is metabolized by a GABA shunt pathway that comprises GABA transaminase (GABA-T), glutamate dehydrogenase (GDH), and succinic semialdehyde dehydrogenase [200]. Jalil et al. [41] have reported that the mutant of *A. thaliana* lacking the GABA-T gene reduces GABA and chlorophyll content, lowers photosynthesis, and GDH activity but increases membrane ion leakage, MDA content, and early leaf senescence under various abiotic stresses. GABA also participates in the signal transduction pathway under stress via the increased cytosolic calmodulin-dependent activity of the enzyme glutamate decarboxylase [36].

It is apparent that the enzymatic and non-enzymatic antioxidants acquire crucial pathways for tight regulation of ROS within plant cells and are responsible for efficient amelioration of abiotic stress-induced oxidative stress. Many researchers have documented the potential role of antioxidants in the alleviation of oxidative stress (Table 2). The tolerant genotypes of *B. juncea* alleviate the heat stress via the increasing activity of enzymatic POX and non-enzymatic GSH antioxidant [201]. Subjection to temperature stress, the tolerant wheat genotypes (HD 2815 and HDR 77) maintain a high activity of antioxidant enzymes SOD, CAT, and APX with the least reduction in the chlorophyll content and lower membrane damage in comparison to that of its susceptible genotypes. The investigation comprehensively establishes alleviating role of antioxidants for the maintenance of structural and functional characteristics in plants [202]. Further, the production of enzymatic antioxidants (SOD, GPX, APX, and GR) with non-enzymatic antioxidants (AsA, GSH) and proline confer tolerance to rice plants against excessive Cu induced oxidative stress [203]. The tolerant lentils to heat stress exhibit elevated SOD and other antioxidants and a negative correlation between MDA and H_2_O_2_, confirming their role in the alleviation of oxidative stress [204]. These studies show that the efficient and coordinated working of antioxidants confer a protective effect on plants under harsh environmental cues.

Plants often encounter multiple stresses simultaneously under field conditions that show a discrete antioxidant activity. For example, *Portulaca oleracea* subjected to combined heat and drought stress exhibits a higher activity of SOD and POX [205]. The cytosolic enzyme APX1 decomposes H_2_O_2_ and plays a significant role in the acclimatization of plants exposed to drought and high-temperature stress concurrently. The *Apx1* deficient mutant of *Arabidopsis* sensitive towards the combined stresses corroborates the findings [206]. Similarly, Zandalinas et al. [190] have observed an enhanced ROS detoxification and resilience to combined heat and drought stress in citrus genotypes Carrizo citrange exhibiting the effective activation of antioxidant machinery. The tolerance ability of Carrizo has been prompting to efficiently coordinate activities of SOD, CAT, APX, and GR with a maintained favorable ratio of GSH/GSSG. While the Cleopatra mandarin subjected to similar stress conditions displays sensitivity due to the increased SOD activity with inefficient activation of GR, diminished CAT activity, and lack of APX activity enhancing oxidative stress.

## 6. ROS as Signaling Molecules

Plants are equipped with an arsenal of adaptive strategies to endure harsh conditions [1,5]. The chief strategy includes initiation of systemic signals from an area under stress to an unstressed region that consequently alerts and activates defense or increases resilience [7,206] arising from signal transducers, including ROS [215]. In addition to oxidative damage, the ROS role has been well recognized as a signaling molecule that prompts tolerance against unfavorable conditions [176]. Inefficient scavenging capacities of antioxidants result in an oxidative burst within plant cells. Under such conditions, the activation or modulation of ROS into signaling transducing pathways could avert damaging consequences of the stress. The imposition of biotic and abiotic stress conditions compels cell organelles to switch the transient ROS production [3,216,217] that offsets ROS homeostasis and initiates the signal transduction cascade [218], involving specific feedback and feed-forward responses facilitating stress tolerance [7]. The spatio-temporal production of ROS is a critical factor that determines the ROS mediated cellular and intracellular signaling [219]. The systemic signaling against ROS generation arises as an auto-propagating wave to an adjacent cell [220] that confers stress tolerance through spatio-temporal communication. For this purpose, plants engage phytohormones and/or amino acids as specific signals to indicate a stress situation [7]. For instance, ROS generated under stress initiate signaling by oxidizing proteins that result in the production of peptides which in turn maintain signaling as a secondary messenger [7]. Among various ROS, H_2_O_2_, a non-ionic, relatively stable yet reactive molecule, diffuses through membranes via aquaporins and initiates signaling. Therefore, H_2_O_2_ acts as a perfect candidate for the signal transduction pathway [7,221]. ROS operates signaling in a highly coordinated manner to regulate stress. It activates antioxidants, kinases, defense genes, the influx of Ca^2+^ ions, protein phosphorylation, increasing synthesis of plant hormones such as salicylic acid, jasmonic acid, ethylene, etc. In the case of biotic stress, it elicits early defense responses such as the synthesis of phytoalexins and pathogenesis-related proteins, as well as cell wall strengthening/PCD promotion, restricting invasion/multiplication/spread of pathogens in plant cells [7,9,176,222]. For instance, GDH that participates in ammonia production/accumulation in stressed cells instead starts synthesizing glutamate and sequentially leading to the production of proline (well known to partake in stress tolerance) in tobacco [7,221]. ROS signaling arbitrates transcription of the gene encoding for GDH α-subunit [7]. ROS such as O_2_**^•^**^–^ and H_2_O_2_ also reportedly participate in plant growth and development, as well as in plant protection against biotic and abiotic stress conditions [3,178]. Therefore, ROS production below the stress threshold induces developmental changes such as the formation of tracheary elements, lignification, and cross-linking in the cell wall leading to PCD and ameliorates abiotic stress [67,223].

The ROS production in cell organelles mediates retrograde signals to the nucleus. The signals move with a speed of 8.4 cm min^−1^ under stress conditions and play a pivotal role as a secondary messenger to alleviate abiotic stress in plants [224,225]. The retrograde signaling assists the nucleus to modulate the anterograde control for the acclimatization of plants exposed to abiotic perturbation [226]. During abiotic stress, the ROS burst elicits the upstream transcription of stress-responsive genes such as heat shock gene (HSG) [225]. HSPs, for example, act as molecular chaperones, partake in the prevention of protein aggregation, misfolding, denaturation, and degradation, as well as facilitate protein refolding particularly during heat stress [225,227]. Apart from heat stress, the role of HSPs in the regulation of light, anoxia, cold, and other abiotic stress has also been documented [225,228]. H_2_O_2_ in the *Arabidopsis* cell culture under heat stress also modulates HSG expression, which induces the production of APX2, HSP17.6, and HSP18.2 [229]. Similarly, the H_2_O_2_ burst in *Arabidopsis* cells upregulates the production of HSPs, APX1 that scavenge H_2_O_2_, and provides tolerance to light stress [230], as well as acclimatizes the plant exposed to the combination of heat and drought stress [206]. The onset of low oxygen stress (hypoxia) consequently leads to the production of ROS in a regulated manner via RBOHs [54]. The regulated production and signaling of ROS are considered an important factor in the management of hypoxic stress [54,225,231,232,233]. Under oxygen deprivation (anoxia/hypoxia stress), H_2_O_2_ upregulates the expression of genes encoding HSPs and genes responsible for fermentation such as *ALCOHOL DEHYDROGENASE,* as well as ROS regulated transcription factor including *ZAT10* and *ZAT12* and proteins, which subsequently facilitate acclimatization to stress [54,225,234,235]. ROS also display systemic signaling in plants via auto-propagation as a wave to adjacent cells [54]. The systemic signaling to neighboring cells by O_2_**^•^**^–^ and H_2_O_2_ in stagnant rice roots and *Arabidopsis*, respectively have been reported [220,224,233]. NADPH oxidase genes viz. *AtrbohF* and *AtrbohD* also trigger the production of ROS during salinity stress that consequently initiates signaling and provide tolerance by regulating Na^+^/K^+^ homeostasis in cells [236]. Jiang et al. [237] have reported that the *soil-salinity sensitive 1-1* mutant of *Arabidopsis* lacking functioning of the NADPH oxidase gene *AtrbohF* does not accumulate ROS in root vasculature and displays hypersensitivity towards salinity stress. Moreover, under nutrient deprivation conditions, ROS induces signaling pathways. ROS in low K^+^ availability upregulates calcium signaling in cells [64,238]. In response to K^+^ deficiency, the H_2_O_2_ concentration increases in plant roots, which enhances the expression of *HAK*5 genes [239]. An understanding and extensive investigation of molecular mechanisms of ROS mediated signaling and cross-talk with other pathways could help develop more tolerant plant varieties that could easily sustain under extremely adverse conditions.

## 7. Strategies and Accomplishments

Improving plants’ ability to adapt and tolerate abiotic stresses under changing climate scenarios is a potential strategy to lessen the oxidative stress-induced damages. Inhibition of pathways that partake in the overproduction of ROS, fortifying the plants’ defense system through recruitment of antioxidants and modulation of ROS into the signaling pathway can boost plant survival under the stressful scenario. Pre-conditioning of plants to non-lethal stress [76] and molecular priming using agents such as micronutrients (β-sitosterol), osmolytes (GABA), etc. can fortify the plant defense mechanism and reduces oxidative stress [36,240,241]. Semwal and Khanna-Chopra [76] have reported that water-deficit pre-conditioning induced the tolerance to subsequent heat stress due to the recovery that escalates activities of antioxidants (SOD, CAT, POX, GSH, DHAR, AsA/DHA ratio, and GSH/GSSH ratio). The exogenous pretreatment with trehalose prompts H_2_O_2_ and the nitric oxide level in tomato leaves under cold stress that mediates signaling, upregulated Cu/Zn SOD, and CAT1 transcripts thereby the enhancement of defense capacity induced tolerance to stress, improvement in growth, and prevention of lipid membrane peroxidation [242]. Analogously, the exogenous application of melatonin in tea and AsA, GSH, and proline in chickpea plants increases the activity of enzymatic antioxidants (SOD, POX, CAT, APX) with the amplified accumulation of GSH and AsA under cold, salt, and/or drought stress [243,244]. The over-reduction of ETC and activities of certain enzymes/redox-active metals are major culprits for ROS overproduction in cell organelles. Consequently, the prevention of ETC over-reduction, inhibiting enzymes, and redox metals can arrest excessive ROS formation. Proline can bind with the redox-active metal ions, thus preventing the production of ^•^OH via the Fenton reaction and safeguard plant cells from oxidative damages [245]. Proline also maintains cellular redox homeostasis by maintaining the NADP^+^/NADPH balance [246]. In the chloroplast, proline is synthesized when glutamate is reduced by NADPH. Consequently, NADP^+^ having been produced prevents the over-reduction of PS I by accepting electrons during stress conditions [94]. In addition, certain compounds such as nitric oxide have been reported to reduce activities of enzymes mediating the ROS production. The activity of XOD in peroxisomes of the *Phalaenopsis* flower that participates in O_2_^•–^ production is downregulated by nitric oxide, leading to alleviation in the ROS level and oxidative stress [247]. Therefore, the priming using potential agents could alleviate ROS overproduction under stress.

Molecular priming is an efficient tool for improving plant tolerance to abiotic stress and its linkage with systems biology can strengthen the potential by unraveling the plants’ complex defense and tolerance mechanism at the molecular level [248]. Systems biology deals with the omics study, i.e., genomics, transcriptomics, proteomics, and metabolomics to understand the functionality of the biological system altogether and facilitates in finding new genes, RNAs, proteins, and metabolites, deciphering their regulatory functions and intracellular interactions [248,249]. Several studies at the molecular level have provided deep insight into the regulatory network controlling response to abiotic stress in plants [250]. Some genes encode for functional proteins or products that directly partake in the regulation of stresses, while some regulate the expression of other stress-responsive genes [251]. The genomic approach focusing on identifying genes encoding for enzymatic antioxidants (APX, GPX, SOD, and CAT) in four resurrection species reveals their major role in the regulation of ROS homeostasis under desiccation (extreme water deficit condition) stress [252]. The study also highlights the ROS detoxification mechanism to be species-specific as having been evidenced through dissimilar expression patterns of all the studied antioxidant gene families. Similar results have been documented by Dubouzet et al. [253] who have reported the expression of dehydration responsive element binding (DREB) transcription factor homolog *OsDREB1A* and *OsDREB1B* gene under low-temperature stress and expression of *OsDREB2A* gene under dehydration and high salinity in rice (*Oryza sativa* L.). The integration of different omics study data elucidates the function and shared pathways of key molecular processes related to the multitude of stress and crops [223] facilitating the development of synthetic biology, which in turn aids in genetic manipulation to develop long-lasting stress-tolerant species [249]. The expression of DREB transcription factor (TF) genes *OsDREB1A* and *OsDREB1B* in transgenic rice improved plant tolerance to drought, high salinity, and cold stresses [254]. These genes encode proteins that might partake in stress tolerance and are associated with an increase in osmoprotectants such as free proline and soluble sugars in transgenic rice plants. A concomitant increase in the amount of osmolytes (free proline and soluble sugars), elevated expression of defense-related genes *OsDREB1A* with enhanced tolerance to drought and salt stress, and alleviated electrolyte leakage have been reported in rice seedlings [251]. Transgenic *Arabidopsis* over-expressing genes encoding for Cu/Zn-SOD demonstrate enhanced resistance against oxidative stress due to the escalated activities of SOD and POD [255]. The over-expression of zinc finger protein gene *OsZFP252* in rice seedlings elevates the expression of defense-related genes *OsDREB1A*, enhances tolerance to drought and salt stress, increases the number of osmolytes (free proline and soluble sugars), and alleviates electrolyte leakage [251]. The indispensable role of *OsZFP252* in the stress-responsive signaling transduction pathway has also been reported. Other studies have also reported the activation of oxidative signaling pathway with an expression of small HSP by the *Nicotiana* protein kinase (NPK1), tobacco mitogen-activated protein kinase kinase kinase (MAPKKK) in transgenic maize that confers protection to photosynthetic machinery after exposure to drought stress [256], and increases tolerance to freezing stress [257].

Recent advances in systems biology have added new impetus to improve plant tolerance. However, the expression and function of RNAs, proteomes, and metabolites in several genotypes are dynamic and still largely unknown. Traditional biotechnological approaches are unable to establish their niche in the field of plant stress management due to hindrance in the translation of identified agronomic traits into phenotypes [250]. The quest to understand the function, interaction, and response of molecular components under an environmental perturbation can be solved by quantifying and characterizing genotype to phenotype relationships [248]. Phenotypic attributes represent a response to abiotic stress. For example, plants growing under excess light possess thick leaves owing to the expanded palisade tissues and high stomatal density, and a lower number of thylakoids per chloroplast compared to plants growing in low light [258]. Phenotypic characters can be studied through a phenomics approach that spans a detailed study of physiological parameters influenced by the plant genetic layout, the spatio-temporal impact of the environment, and agricultural management practices [259]. The non-invasive method using cameras and sensor-based imaging (fluorescence, visible light, and infrared imaging, X-ray computed tomography, etc.) and advanced instruments such as fluorometers together with robust software systems are emerging techniques for studying plant morphological and developmental responses under a prevalent environment [258,259,260]. Such techniques enable us to explore information regarding the chemical composition and function that can be accessed from the cell to plant canopy level [260]. Under stress, the measurement of green and yellow areas of the leaf facilitates the determination of leaf senescence and tissue tolerance, corresponding to salt accumulation [260]. Similarly, studying chlorophyll fluorescence to monitor the impact of abiotic stress on plant photosynthesis and overall performance is a potential phenotyping technique [258]. Furthermore, chlorophyll fluorescence may be used to detect acclimatization mechanisms among genotypes under a defined set of stresses [258]. In particular, the variation in NPQ and leaf development has been observed in different accessions of *Arabidopsis* under similar environmental conditions [258]. In addition to the amalgamation of systems biology with plant physiology, crop modeling approaches further outline the plant responses by designing multiple simulations for farming practices and predicted climate change [261]. Crop models are expected to assist extrapolation of the complexity of climate change [261]. The climate-resilient barley (*Hordeum vulgare* L.) ideotypes designed through an assemblage of eight barley simulation models for the boreal and Mediterranean climate have revealed that specific ideotypes with a particular set of traits such as longer reproductive growing phase, higher radiation use efficiency/maximum assimilation rate, lower leaf senescence rate, and drought tolerance, in addition to a long (for boreal climate)/short (Mediterranean climate) photoperiod and vernalization sensitivity makes them promising cultivars for a projected future climate compared to other genotypes and confers better yield with a lower inter-annual yield variation [262]. A well-spun amalgamation of new and advanced scientific technologies could generate vast information that can be exploited to improve the plant tolerance capacity and resilience against hostile situations.

## 8. Conclusions and Future Prospects

Intensified abiotic stresses have perturbed ecological fitness via the production of ROS in plant cells. In the coming decades, the crisis will aggravate, challenging the plants’ survival. ROS are similar to a double-edged sword that induces oxidative stress in plants when their production exceeds threshold levels, but at low or moderate concentrations, mediates the signal transduction that assists in maintaining cellular homeostasis and facilitates plant acclimatization to stress(es). To maintain equilibrium between ROS generation and their quenching, plants recruit antioxidants. Nevertheless, their potential diminishes during stress. Devising techniques that could avert damaging aspects of ROS under stress conditions and improving plants’ tolerance mechanisms can unlock avenues for designing new generation stress-resilient crops. Recently, the techniques such as molecular priming or pre-conditioning have demonstrated the immense potential to improve plant resistance against abiotic stresses, however, some gaps still exist. Molecular priming requires a particular timing for the application of priming agents, for instance, just before the onset of stress, thus it requires continuous monitoring of environmental conditions to fortify plants against stress at both local and global levels. Furthermore, molecular priming has shown promising results in hydroponic or controlled conditions but the estimation of their efficiency in a real field under a present and projected climatic scenario is a necessity. In addition, the elucidation of pathways and impact of cross-talk between different priming agents and cellular compounds such as signaling agents, phytohormones, etc., within plant cells is important to gain insight into the fate of priming agents under variable conditions.

**Figure 8:**
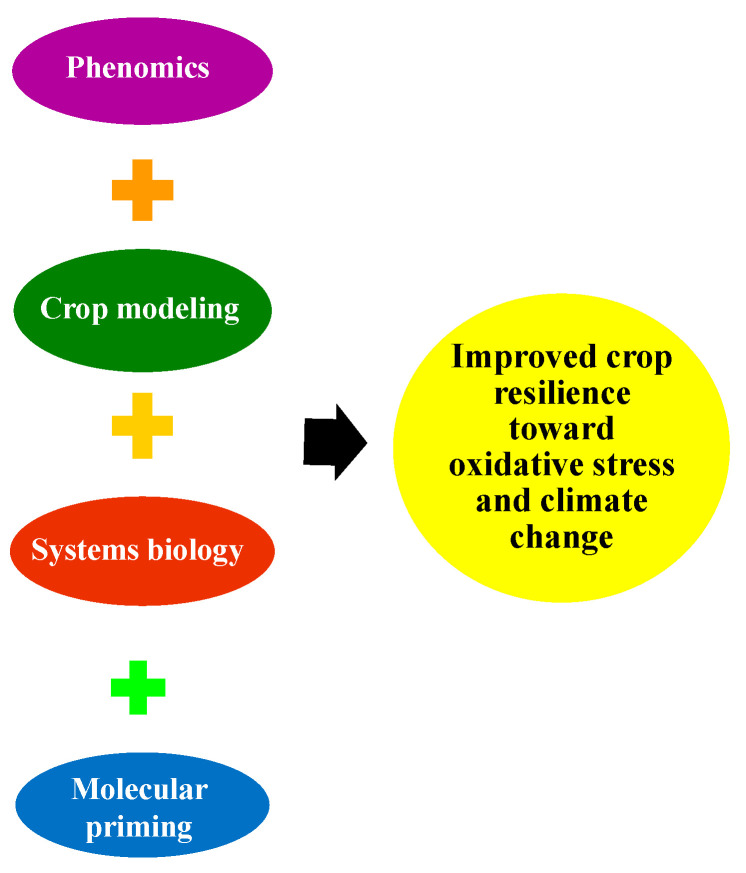
Integration of systems biology, phenomics, crop modeling, and molecular priming as a holistic approach for the development of climate-smart crops to improve growth and productivity.

To fill the gaps, the integration of various disciplines such as systems biology, phenomics, and crop modeling with molecular priming is required (Figure 8). The identification of key genes, transcripts, proteins, and metabolites governing multiple pathways (signaling as well as the oxidation of biomolecules) through the systems biology approach assist in the discovery of new avenues. For example, the identification of genes responsible for the synthesis of priming agents endogenously in accordance with changing environmental conditions could eliminate the problem of continuous environmental monitoring at a global scale. Therefore, there is a need for intensive and dedicated research for the development of resilient crops via the application of molecular tools such as QTL mapping for the identification of the genomic network of metabolite biosynthesis and genome editing tools such as the regularly clustered interspaced short palindromic repeats and CRISPR-associated proteins (CRISPR/Cas). In addition to deciphering and improving the plant genetic network and underlying mechanisms, their vigilant amalgamation with phenomics and crop modeling is also necessary to maintain the unabridged potential of crops, while maintaining the ecosystem sustainability under climate change scenarios. The molecular responses of plants are highly influenced by environmental dynamics and demonstrate unique characters under each set of conditions. Therefore, quantification of the relationship between the genotype and phenotype under changing environmental conditions is important. This could be achieved through an integration of phenomics and crop modeling studies that furnish data related to the behavior of a particular or set of genetic networks in consonance to the dynamic environment. This integrated research framework could be highly obliged for laying out new improved management techniques that sustainably lead to the development of climate-smart crop cultivation with long-lasting tolerance to oxidative stress and boosts economical productivity.

## Figures and Tables

**Table 1:** Abiotic stresses triggered secondary stresses and their damaging effects on plant growth and activity.

Abiotic Stress	Induced Secondary Stresses	Effects in Plant	References
Chilling/freezing stress	Nutritional imbalance, osmotic and oxidative stress	Accumulation of ROS and oxidative damage; inhibition of enzymes’ activities and metabolic imbalance. Increased cell dehydration and starvation, senescence, delayed maturation, damage of PS II, and reduced photosynthetic activity. Decreased growth and productivity.	[20,21]
Drought	Osmotic, heavy metal, and oxidative stress	Increased ROS production and ion leakage; induced dehydration and turgor loss. Decrease in absorption and translocation of mineral nutrients. Protein denaturation, loss of enzyme activities, reduced photosynthetic activity due to abridged chlorophyll content and CO_2_ assimilation. Increase in leaf temperature, premature abscission, necrosis, and stunted plant growth.	[22,23]
Flooding/waterlogging	Water and nutrient deficiency stress, oxidative stress	Increased ROS and ethylene production and decreased antioxidants level. Reduced stomatal conductance; abridged water and nutrient uptake. Reduced gaseous exchange, anoxia/hypoxia, increased anaerobic metabolism and inhibited root respiration; reduced photosynthetic activity due to the decreased chlorophyll content and damage of PS II. Stunted growth and senescence of leaf and inflorescence.	[24,25]
Heat stress	Water scarcity, osmotic and oxidative stress	Enhanced ROS production and oxidative damage, protein misfolding, and denaturation. Growth inhibition, foliar senescence, and abscission, leaf and fruit discoloration, reduced CO_2_ fixation, PS I and PS II disruption, disturbed ion transport.	[26,27,28]
Heavy metals/xenobiotic compounds	Nutrient and oxidative stress	Increased ROS production and oxidative damage. Disruption of function and structure of enzymes; reduced stomatal conductance, CO_2_ assimilation, and net photosynthesis rate. Reduced biomass accumulation, inhibition of seed germination, and impaired nutrient uptake.	[29]
Light/radiation stress	Oxidative stress	Increased ROS production and oxidative damage, disrupted photosynthesis ETC, and/or increased activity of membrane-bounded NADPH oxidase, chlorophyll degradation, reduced photosynthetic activity, and epidermal cell expansion inhibition. Leaf senescence, reduced rosette diameter, condensed inflorescence stem with a boosted number of flowering stems.	[30]
Nutrient imbalance	Oxidative stress	ROS accumulation with reduced antioxidants; increased leakage of ion and solutes, reduced activities of metalloenzymes, declined photosynthesis. Susceptibility to other biotic and abiotic stresses. Stunted growth, chlorosis, necrosis, poor flowering and fruiting, declined productivity.	[31,32]
Ozone (O_3_) stress	Oxidative stress	ROS production inducing oxidative damage, inhibited enzyme activities, chlorophyll and xanthophyll degradation, diminished stomatal conductance, and decreased photosynthesis. Leaf chlorosis and necrosis, early senescence, and reduced plant biomass and productivity.	[33,34]
Salinity	Water scarcity, ionic imbalance, nutrient, osmotic and oxidative stress	ROS production causing oxidative damage, restricted uptake and translocation of water and mineral nutrients causing Na^+^ toxicity and decreased K^+^, Ca^2+^, and Mg^2+^ content, reduced soil water potential. Decreased stomatal opening, disorganized thylakoid ultrastructure, and reduced photosynthesis. Reduced seed germination, immature leaf senescence, and abridged growth and productivity.	[7,35,36]

**Table 2:** Antioxidant activity in plants in response to abiotic stress-induced oxidative stress.

Abiotic Stress(es)	Plant Exposed	Antioxidant(s) Activity	References
Chilling stress	*Cucumis sativus* (Cucumber)	The activity of SOD, APX, GR, and GP increased and CAT activity decreased in leaves.	[207]
Chilling stress	*Zea mays* (Maize) seedling	Exogenous application of nitric acid before the onset of stress increased the activity of SOD and POX. ROS level and lipid peroxidation alleviated.	[20]
Drought	*Triticum aestivum* (Wheat)	The upregulated APX and balanced redox pool of AsA and GSH fortified photosynthetic apparatus and mitochondria in acclimatized plants.	[208]
Heavy metal (Cu) stress	*Oryza sativa* (Rice)	The activity of SOD, guaiacol peroxidase (GP), APX, GR, AsA, GSH with proline increased. CAT activity remained unaltered. H_2_O_2_ level and lipid peroxidation declined.	[203]
High-temperature stress	*Triticum aestivum* (Wheat)	The activity of SOD, APX, CAT, POX, and GR increased in tolerant genotype C306.	[209]
High-temperature stress	*Spinacia oleracea* (Spinach) seedling	Overexpression of the gene encoding cytosolic heat shock 70 protein (SoHSC70) increased the activity of SOD, POX, CAT, and APX enzymes. Oxidative membrane damage and ROS accumulation reduced.	[210]
Metalloid (Boron) stress	*Artemisia annua*	The activity of SOD, POX, and CAT increased.	[211]
Salinity stress	*Oryza sativa* (Rice) seedling	Exogenous application of manganese to seedlings exposed to stress increased non-enzymatic antioxidants (phenolic compounds, flavonoids, and AsA), and enzymatic antioxidants (MDHAR, DHAR, SOD, and CAT) content. ROS level reduced.	[35]
UV-B radiation	*Helianthus annuus* (Sunflower) cotyledons	The activity of CAT, glutathione dehydrogenase, GP, and the ratio of GSH/GSSG increased. The AsA/DHA ratio, APX, and GR activity remained unaltered. Lipid peroxidation and oxidative damage in cotyledons reduced.	[212]
Low temperature + herbicide (isoproturon) stress	*Triticum aestivum* (Wheat) seedling	Foliar application of AsA increased activity of antioxidants SOD, CAT, and POX. MDA content and ROS production rate declined.	[213]
Salinity + herbicide (2,4 dichlorophenoxyacetic acid) stress	*Oryza sativa* (Rice)	Enzymatic (SOD, CAT, APX, and POX) and non-enzymatic (phenolic compounds, total soluble phenols, proline, and sugars) antioxidants level modulated. H_2_O_2_ and O_2_^•–^ content decreased; oxidative stress and lipid peroxidation alleviated.	[214]
